# *Toxoplasma gondii* in livestock in St. Kitts and Nevis, West Indies

**DOI:** 10.1186/s13071-015-0776-7

**Published:** 2015-03-18

**Authors:** Clare M Hamilton, Patrick J Kelly, Paul M Bartley, Alison Burrells, Alice Porco, Deidra Metzler, Kirsten Crouch, Jennifer K Ketzis, Elisabeth A Innes, Frank Katzer

**Affiliations:** Moredun Research Institute, Pentlands Science Park, Bush Loan, Edinburgh, EH26 0PZ UK; Ross University School of Veterinary Medicine, PO Box 334, Basseterre, St. Kitts West Indies

**Keywords:** *Toxoplasma gondii*, Livestock, Abattoir, Caribbean, Seroprevalence, Genotype

## Abstract

**Background:**

*Toxoplasma gondii* is a ubiquitous protozoan parasite capable of infecting all warm-blooded animals including livestock. In these animals, the parasite forms cysts in the tissues which may pose a risk to public health if infected meat is consumed undercooked or raw. The aim of this study was to determine the exposure of livestock to *T. gondii* in St. Kitts and Nevis.

**Methods:**

Sera and/or heart tissue and meat juice were collected from pigs (n = 124), sheep (n = 116) and goats (n = 66) at the St. Kitts Abattoir. Sera and meat juice were screened for reactive antibodies to *T. gondii* using an in-house ELISA. Heart tissue was screened for *T. gondii* DNA using quantitative PCR and positive samples were genotyped using RFLP.

**Results:**

Antibodies to *T. gondii* were detected in sera from 48% of pigs, 26% of sheep and 34% of goats tested. Antibodies were also detected in the meat juice from 55% of pig hearts, 22% of sheep hearts and 31% of goat hearts tested. There was a significant positive correlation between serology and meat juice results. *T. gondii* DNA was detected in heart tissue of 21% of pigs, 16% of sheep and 23% of goats tested. Preliminary PCR-RFLP analysis identified a predominance of the Type III genotype of *T. gondii*.

**Conclusions:**

These results suggest widespread environmental contamination with *T. gondii* oocysts and that livestock could be a potentially important source of *T. gondii* infection if their infected meat is consumed (or handled) undercooked.

## Background

*Toxoplasma gondii* is a ubiquitous protozoan parasite capable of infecting all warm-blooded animals, including people [1]. Felids are the only known definitive host of the parasite and can shed millions of environmentally resistant oocysts in their faeces following primary infection [2]. In intermediate hosts, the parasites develop into cysts in various tissues and may persist in a viable state for the lifetime of the host. Most infections of herbivorous livestock follow ingestion of infective oocysts contaminating the pasture, feeds or drinking water. Infection of pigs can also occur this way or through the ingestion of rodents or other small mammals harbouring *T. gondii* cysts in their tissues [3]. Congenital transmission, resulting from a primary infection with *T. gondii* during pregnancy, can occur in most livestock and is a major cause of reproductive failure in sheep and goats worldwide. Although there are occasional abortions and premature births in pigs, most infections are subclinical or result in mild, non-specific signs. Cattle very rarely exhibit clinical signs [4].

Worldwide seroprevalences of *T. gondii* in livestock vary widely, ranging from 3% to 96% in sheep [5], 4% to 77% in goats [6], 0.4% to 96% in pigs [7,8] and 2% to 83% in cattle [6,9], with seropositivity increasing with age [10]. Once infected, livestock may harbour *T. gondii* tissue cysts for the duration of their lifetime, presenting a potentially significant risk to public health if their meat is consumed raw or undercooked. It is estimated that one third of the human population is infected with *T. gondii* although regional seroprevalences vary widely [11]. Humans become infected with *T. gondii* by ingesting tissue cysts from meat, or by ingesting oocysts from contaminated food or water, or directly from the environment. The importance of transmission routes in humans may vary between different ethnic groups and geographical locations; however, consumption of undercooked meat is a significant risk factor and may result in 50% or more of toxoplasmosis cases [12]. In immune-competent people, toxoplasmosis is usually subclinical or a mild, flu-like disease; however, in immune-compromised individuals, there can be severe clinical signs and fatalities [13]. Congenital toxoplasmosis can lead to abortion, neonatal death, neurological signs such as hydrocephalus, or ocular signs such as chorioretinitis [13]. The disease burden of congenital toxoplasmosis, as represented by the disability-adjusted life years, is the highest among all food-borne pathogens [12].

Variation in disease outcome may be related to inoculum dose, infecting stage, and the genetic diversity of the infecting strain [6]. Previously, *T. gondii* was thought to comprise 3 predominant clonal lineages (designated Types I, II and III), with little genetic diversity [14,15]. Recent reports from Brazil and French Guiana, however, have documented cases of severe toxoplasmosis and ocular disease in immune-competent patients following infection later in life. Disease in these individuals has been linked to genetically distinct strains of *T. gondii* [16,17].

Although the more limited and distinct geography and biodiversity of the Caribbean islands facilitates epidemiological studies on *T. gondii*, there is little data on infections. Infections appear to be common, however, with seroprevalences of 8% to 43% reported in livestock from various islands [18,19]. Furthermore, there are high seroprevalences in domestic (85%) and feral (74%) cats [20,21] on St. Kitts, and genetic characterization of isolates from some of the feral cats revealed 4 genotypes, including Type II, Type III and two unique genotypes [21]. Genetic characterization of *T. gondii* isolates from chickens in Grenada revealed a predominance of Type III [19], and a recent study in dogs on the island reported the presence of unique genotypes along with Types II and III [22].

To provide further information on *T. gondii* in the Caribbean, we performed serology on livestock being slaughtered at the St. Kitts Abattoir and used real time PCR to detect parasite DNA within their tissues.

## Methods

### Sampling location and animals

Saint Kitts and Nevis are a small island federation located in the Eastern Caribbean, 17° 20’ North, 62° 45’ West. St. Kitts is 168 km^2^ with a population of approximately 35 000, and Nevis is 93 km^2^ with a population of approximately 15 000. St. Kitts Abattoir is located in Basseterre, the capital of St. Kitts, and processes sheep, goats, pigs and cattle from both islands. The majority of animals are brought to the abattoir by traders who have bought them from farmers on either/both island. As the varying numbers of animals brought to the abattoir each week by traders often came from multiple farms, it was not possible to accurately determine the demographics of the animals. Cattle were excluded from the study as they appear to play little role in the epidemiology of human toxoplasmosis and only small numbers (0-7) were slaughtered each week. Sheep belonging to Ross University School of Veterinary Medicine (RUSVM), St. Kitts, were also used in the study. Demographic data on these animals was also not available as they originated from farms around St. Kitts.

The study was conducted between November 2013 and March 2014. Ethical approval was obtained from the Institutional Animal Care and Use Committee of RUSVM, and permission for collection of samples at St. Kitts abattoir was granted by St. Kitts and Nevis Government and the Chief Veterinary Officer. Permission was also sought from the traders by the abattoir manager.

### Antigen preparation for ELISA

*Toxoplasma gondii* RH strain tachyzoites were grown in pre-cultured Vero cells (ATCC® CCL-81™) in IMDM (supplemented with 2% FCS, 200 IU penicillin/ml and 200 μg/ml streptomycin) at a ratio of 1:2 (Vero cell: *T. gondii* tachyzoites). When all Vero cells were lysed and free tachyzoites were visible in the medium, it was centrifuged for 10 min at 675 × g and the supernatant discarded. After two washes in 50 ml PBS (675 × g for 10 min) the pellet was resuspended in 25 ml PBS and tachyzoites counted using a Neubauer haemocytometer. After adjusting the final concentration to 10^9^ tachyzoites per ml with antigen extraction buffer (50 mM sodium phosphate pH 7.6, 1 mM phenylmethanesulfonylfluoride (PMSF), 2 mM EDTA pH 7.5, 2 μg/ml pepstatin), saponin (0.5%) and octylglucoside (0.5%) were added and the suspension was incubated for 16 h at 4°C on a blood tube rotator. Following centrifugation for 30 min at 50,000 × g at 4°C, antigen concentration in the supernatant was measured using a Pierce BCA Protein Assay Kit (ThermoFisher, Northumberland, UK). Aliquots (500 μl) of supernatant were stored at -80°C until use.

### Sera and meat juice preparation

Blood samples were collected directly from the hearts of pigs, sheep and goats, following removal after slaughter at St. Kitts Abattoir, using 18G disposable needles into vacuum tubes without anti-coagulant. Further blood samples were collected from the sheep being held at Ross University School of Veterinary Medicine, St. Kitts. All sera were obtained following centrifugation (2000 × g for 10 min at 4°C) of clotted blood samples and stored at -20°C until use.

Meat juice (fluids that leaked from heart tissue (Section 2.5.1), following 24 h storage at 4°C) was centrifuged at 1000 × g for 2 min to remove debris and supernatants stored at -20°C until use.

Samples were collected from 124 pigs, 116 sheep and 66 goats (Table 1). A complete set of samples was not always obtained from each animal. In some cases we obtained sera alone as trader permission to remove heart tissue, and hence meat juice, could not be obtained (15 pigs, 5 sheep and 2 goats). In other cases, we obtained only heart tissue and meat juice. This occurred when blood could not be aspirated from hearts damaged when carcasses were split (21 pigs, 11 sheep, and 2 goats). We obtained only sera form the 35 live sheep kept at RUSVM.

**Table 1:** **Samples used in the study and their source**

**Sample description**	**Pigs**	**Sheep**	**Goats**
Sera, meat juice and heart tissue	88	65	62
Sera only	15	40	2
Heart tissue and meat juice only	21	11	2

### *Toxoplasma gondii* ELISA

In-house ELISAs were adapted from the methodologies of Buxton *et al*. [23] for sheep and goat sera, and from Burrells [24] for pig sera. Microwells of Greiner Bio-One 96-well medium binding plates were coated with 100 μl/well of solubilised RH antigen (See section 2.2) at a concentration of 3 μg/ml in 0.05M sodium carbonate buffer (pH 9.6) and incubated at 4°C overnight. Following incubation, plates were washed 3 times with PBS (pH 7.2) containing 0.05% Tween-20 (PBST).

Sheep and Goat ELISA: Control sera, test sera and meat juice were diluted 1:500 in 1% BSA in PBST (BSA/PBST) and 100 μl was added to the appropriate microwells, in duplicate. Plates were incubated for 2 h at 37°C, and washed 3 times in PBST. 100 μl HRP-conjugated Protein G (Invitrogen) diluted 1:20,000 in 1% BSA/PBST was added to each well and plates were incubated for 2 h at 37°C. Plates were washed 3 times in PBST, and 100 μl substrate (TMB) was added to each well and incubated for 25 min at room temperature. The reaction was stopped by the addition of 2M H_2_SO_4_. The OD of each plate was measured at 450 nm using a microplate reader. Duplicate samples of positive and negative control serum were included on each plate. Control sera were pooled samples from 5 sheep experimentally infected with *T. gondii*, and 5 negative control sheep from the same experiment [25].

Pig ELISA: Plates were blocked for 1 h at 37°C with 125 μl/well 1% BSA/PBST, and washed 3 times in PBST. Control sera, test sera and meat juice were diluted 1:100 in 1% BSA/PBST and 100 μl was added to the appropriate microwells, in duplicate. Plates were incubated for 1 h at 37°C, and washed 3 times in PBST. 100 μl HRP-conjugated Protein G (Invitrogen) diluted 1:20,000 in 1% BSA/PBST was added to each well and plates were incubated for 1 h at 37°C. Plates were washed 3 times in PBST, and 100 μl substrate (TMB) was added to each well and incubated for 25 min at room temperature. The reaction was stopped by the addition of 2M H_2_SO_4_ and the OD was measured at 450nm using a microplate reader. Duplicate samples of negative and positive control serum were included on each plate. Control sera were pooled samples from 5 pigs experimentally infected with *T. gondii*, and 5 negative control pigs from the same experiment [24].

For each plate, the cut-off value was calculated as two times the percent positivity of the negative control serum relative to the positive control serum (i.e. [2 × (average negative control sera OD/average positive control sera OD)] × average positive control) [26].

### Detection of *T. gondii* DNA in livestock

#### Collection of heart tissue

Heart tissue was collected once the pluck (heart, liver, windpipe and lungs) had been removed from slaughtered pigs, sheep and goats by abattoir staff. Portions of the left and right ventricles (at least 50 g) were removed and stored individually in sterile sample pots for transportation to the laboratory at RUSVM. Between each sample, knives were washed in water and wiped dry to minimise cross-contamination.

#### Pepsin digest and DNA extraction

All heart tissues were digested with acid-pepsin as described by Dubey [27], with modifications. Briefly, 50 g tissue per sample (free of connective tissue and fat) was cut into 1-2 cm pieces and homogenised in a 1 L glass beaker for 30 sec using a hand-held blender. After 100 ml saline (0.9%) was added to the sample it was homogenised again for 30 sec and transferred to a 500 ml glass bottle. The blender and beaker were rinsed with 100 ml saline and these washings were added to the homogenate along with 250 ml pre-warmed (37°C) acid-pepsin solution. After incubation for 60 min at 37°C, with frequent mixing, the homogenate was filtered through two layers of gauze, and the resulting filtrate centrifuged for 10 min at 1200 × g. The pellet was resuspended in 20 ml PBS and neutralized with 15 ml 1.2% sodium carbonate before centrifugation for 30 min at 2000 × g. Two millilitres of the homogenised pellet was taken for DNA extraction using the Wizard® genomic DNA purification protocol (Promega Corporation, U.K). Volumes used in the manufacturer’s protocol were up-scaled to allow for the larger starting material, and the final DNA pellet was resuspended in 200 μl DNase/RNase-free water and stored at -80°C prior to PCR analysis. To monitor potential cross-contamination between samples, extraction controls (using DNase/RNase-free water) were included within each batch of DNA extractions and processed identically to homogenised tissue.

#### Real-time quantitative PCR on 529-bp repeat element

PCR amplifications were carried out, in triplicate, according to the method developed and described by Opsteegh *et al.* [28], with slight modifications. Amplifications were performed in 96-well plates using an Applied Biosystems 7500 Fast Real Time PCR System. The 20 μl reaction mixture consisted of: 10 μl 2X TaqMan Fast Universal PCR Mastermix No AmpErase® UNG (Applied Biosystems), 0.7 μM of each primer (see [28] for sequences), 0.1 μM Tox-TP1 probe (see [28] for sequence) labelled with 6-FAM (5′ end) and black hole quencher (BHQ; 3′ end), 0.2 μM CIAC probe (see [28] for sequence) labelled with JOE (5′ end) and BHQ (3′ end), 0.01 fg competitive internal amplification control (CIAC; See [28] for details), and 250 ng template DNA in 5 μl. The reaction mixture was initially incubated at 95°C for 10 min to activate hot-start DNA polymerase. This was followed by 45 amplification cycles that consisted of a 10 s denaturation step at 95°C, an annealing step at 58°C for 20 s, and an extension step at 72°C for 32 s. Fluorescence was measured at 530 nm (Tox-TP1) and 560 nm (CIAC-probe) at the end of each extension step. A *T. gondii* standard series was included in each run for calculation of the standard curve. Non-template controls (NTC) and extraction controls were included in each run. Samples which came up positive on the qPCR were electrophoresed on a 3% agarose gel incorporating Biotium GelRed™ (Cambridge Bioscience Ltd, U.K) to confirm the size of amplicons.

#### Genetic characterisation of *T. gondii* by multiplex nested PCR-RFLP

All samples that tested positive by qPCR were initially genotyped using PCR-RFLP genetic markers SAG2 (3′ and 5′), SAG3, BTUB and GRA6 [29-31], using multiplex nested PCR conditions as previously described [32]. Samples that were positively genotyped at one or more loci were further genotyped using genetic markers SAG1, C22-8, C29-2, L358, PK1 and Apico [29,31]. The multiplex PCR reaction for the latter markers was carried out in a 20 μl reaction volume containing 2 μl 10x custom PCR mix [32], 0.1 μM of forward and reverse external primers for each of the markers [29], 0.75 units BioTaq (Bioline), 5.9 μl DNase/RNase-free dH_2_O and 2 μl DNA. To improve the sensitivity of the technique, each multiplex reaction was carried out in quadruplicate. First round cycling conditions were 4 min at 95°C, followed by 25 cycles of 30 sec at 94°C, 1 min at 53.7°C, and 2 min at 72°C. The nested PCR reaction used separate internal primers for each of the markers [29], and used the first round PCR products diluted 1:1 in dH_2_O instead of DNA. The nested PCR was carried out in a 20 μl reaction volume containing 2 μl 10x custom PCR mix (as above), 0.3 μM of forward and reverse primers for each of the markers (except SAG3 where 0.1 μM of forward and reverse primers were used), 0.75 units BioTaq (Bioline), 13.9 μl DNase/RNase-free dH_2_O and 2 μl diluted first round PCR product. Second round cycling conditions were 4 min at 95°C, followed by 40 cycles of 30 sec at 94°C, 1 min at 60°C, and 1.5 min at 72°C [32]. Positive controls and NTC were included in each PCR run.

Positive nested PCR products (3 μl) were digested with the appropriate restriction enzymes as previously described [29,31]. Fragments were separated on a 3% Metasieve agarose gel (Flowgen Bioscience Ltd, U.K) incorporating Biotium GelRed™, and typing was based on RFLP patterns of control reference strains, B1 (Type I), M4 (Type II) and NED (Type III) [32].

#### Statistical analysis

Correlation between serum ELISA ODs and meat juice ELISA ODs was carried out using concordance correlation coefficient. Level of agreement between serology results and qPCR results were investigated using Cohen’s kappa coefficient. A *P* value of < 0.05 was deemed significant. All statistical analyses were carried out using the statistical software package, SPSS.

## Results

### Seroprevalence of *T. gondii* in livestock animals

**Figure 1:**
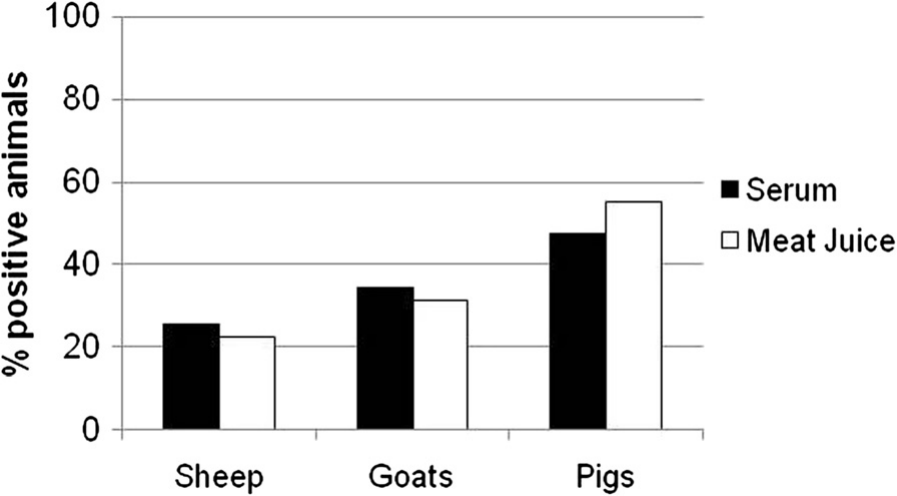
**Seroprevalence of** ***Toxoplasma gondii*** **in livestock in St. Kitts and Nevis.**

Antibodies to *T. gondii* were detected in 48% (95% confidence interval (CI): 38-57%) of pig sera (49/103), 26% (CI: 18-35%) of sheep sera (27/105) and 34% (CI: 24-47%) of goat sera (22/64) tested by ELISA (Figure 1). Meat juice was also screened for *T. gondii* antibodies to determine its potential as an alternative test sample to sera. Antibodies were detected in 55% (CI: 47-65%) of pig meat juice samples (60/109), 22% (CI: 14-33%) of sheep meat juice samples (17/76) and 31% (CI: 21-44%) of goat meat juice samples (20/64) tested by ELISA (Figure 1). For 215 animals (88 pigs, 65 sheep and 62 goats), there was both a serum ELISA result and a meat juice ELISA result. For these samples, Lin’s concordance correlation indicated a significant positive association between serum ELISA ODs and meat juice ELISA ODs (rho_c = 0.899, *P* < 0.001).

### Detection of *T. gondii* in tissues of livestock and correlation with serology

Overall, 20% (50/249) of the hearts tested were positive for *T. gondii* DNA. Results differed between species, with 21% (CI: 14-30%) of pig hearts testing positive (23/109), 16% (CI: 9-26%) of sheep hearts (12/76) and 23% (CI: 15-35%) of goat hearts testing positive (15/64) (Table 2).

**Table 2:** **Detection of** ***Toxoplasma gondii*** **DNA in livestock tissues**

**Livestock species**	**n tested**	**n positive**	**% positive**
Pigs	109	23	21.1
Sheep	76	12	15.8
Goats	64	15	23.4

There was matching serum ELISA results for 215 of the 249 hearts screened for *T. gondii* DNA (Table 3). Similar percentages of pigs (21%; 18/88), sheep (15%; 10/65) and goats (23%; 14/62) were positive by both qPCR and serum ELISA whereas a relatively high percentage of pigs (32%; 28/88) were positive by serum ELISA yet negative by qPCR, compared with only 11% of sheep (7/65) and goats (7/62). Only one goat (2%; 1/62) and two pigs (2%; 2/88) were positive by qPCR and yet negative by serum ELISA. Cohen’s kappa coefficient demonstrated there was a moderate (*k* = 0.5204) level of agreement between positive serum ELISA results and positive qPCR results.

**Table 3:** **Correlation between serum ELISA results and qPCR results (n = 215)**

**Livestock species**	**n**	**ELISA**	**qPCR**		**Total**
			**Pos**	**Neg**	
Pigs	88	Pos	18	28	46
		Neg	2	40	42
Sheep	65	Pos	10	7	17
		Neg	0	48	48
Goats	62	Pos	14	7	21
		Neg	1	40	41
Total	215		45	170	215

Meat juice samples were obtained from all 249 hearts screened for *T. gondii* DNA by qPCR and all were screened for antibodies by ELISA (Table 4). Numbers of animals that were both qPCR and serologically positive were similar to those above where sera was used for ELISA - 20% of pigs (22/109) and goats (13/64), and 16% of sheep (12/76). This was also the case for animals that were positive by meat juice ELISA but negative by qPCR - 35% of pigs (38/109), 11% of goats (7/64) and 7% of sheep (5/76); and for animals positive by qPCR but negative by meat juice ELISA – 3% goats (2/64) and 1% pigs (1/109). Again, Cohen’s kappa coefficient demonstrated a moderate (*k* = 0.5030) level of agreement between positive meat juice ELISA and positive qPCR results.

**Table 4:** **Correlation between meat juice ELISA results and qPCR results (n = 249)**

**Livestock species**	**n**	**ELISA**	**qPCR**		**Total**
			**Pos**	**Neg**	
Pigs	109	Pos	22	38	60
		Neg	1	48	49
Sheep	76	Pos	12	5	17
		Neg	0	59	59
Goats	64	Pos	13	7	20
		Neg	2	42	44
Total	249		50	199	249

### Genetic characterisation of *T. gondii*

Of the 50 animals that tested positive for *T. gondii* by qPCR, 20 (40%) were successfully amplified with PCR-RFLP primers for 1 or more *T. gondii* markers across 4 loci (Table 5). Of the 20 *T. gondii* genotypes amplified, 13 originated from pigs, 4 from sheep and 3 from goats. Genotyping revealed that the predominant lineage across all species was Type III (55.0%), with 2 animals (both pigs) displaying this genotype across all 5 markers (animals 99 and 123) (Table 5). Two animals (both pigs) displayed Type I genotypes – one animal (9) displayed this genotype across all 5 markers, the other (69) was only amplified at a single locus (BTUB). Although no animals displayed the Type II genotype, some had genotypes with apparent re-assorted markers (36 and 37 (both sheep); 97 (pig)). One animal (91; pig) appeared to display a mixed infection with Type II and Type III alleles present at the BTUB locus. Three animals (195, 203, 209; 2 goats and 1 pig, respectively) only amplified at the 5’SAG2 locus where a Type I or Type II genotype could not be deciphered. Attempts to amplify products at additional loci in these animals failed.

**Table 5:** **Genotyping of** ***Toxoplasma gondii*** **DNA isolated from heart tissue from livestock animals in St. Kitts and Nevis**

		**PCR-RFLP marker**					
**Reference/Sample**	**Species**	**5′SAG2**	**3′SAG2**	**SAG3**	**BTUB**	**GRA6**	**Genotype**
B1 – Type I		I or II	I or III	I	I	I	I
M4 - Type II		I or II	II	II	II	II	II
NED - Type III		III	I or III	III	III	III	III
6	Pig	Na	Na	III	Na	Na	III^a^
9	Pig	I or II	I or III	I	I	I	I
10	Pig	Na	Na	III	III	III	III
23	Pig	III	I or III	III	Na	III	III
29	Pig	III	Na	Na	Na	Na	III^b^
36	Sheep	I or II	II	II	Na	I	Re-assort. I and II^d^
37	Sheep	Na	Na	II	Na	I	Re-assort. I and II^d^
69	Pig	Na	Na	Na	I	Na	I^c^
72	Pig	III	I or III	III	Na	Na	III
83	Sheep	III	Na	Na	Na	Na	III^b^
91	Pig	I or II	II	III	II and III	II	Mixed II and III
97	Pig	I or II	II	III	Na	Na	Re-assort. II and III^d^
98	Pig	Na	Na	III	Na	III	III
99	Pig	III	I or III	III	III	III	III
123	Pig	III	I or III	III	III	III	III
136	Goat	III	I or III	III	Na	Na	III
158	Sheep	III	I or III	III	Na	Na	III
195	Goat	I or II	Na	Na	Na	Na	I or II^b^
203	Goat	I or II	Na	Na	Na	Na	I or II^b^
249	Pig	I or II	Na	Na	Na	Na	I or II^b^

Na = no amplification.

^a^Based on a single allele at the SAG3 locus.

^b^Based on a single allele at the 5′SAG2 locus.

^c^Based on a single allele at the GRA6 locus.

^d^Possible re-assorted markers.

## Discussion

Relatively little is known about the epidemiology of *Toxoplasma gondii* in the Caribbean. Our findings demonstrate that pigs, sheep and goats on St. Kitts and Nevis are exposed to *T. gondii* in their environment*.* Previous studies on livestock have reported seroprevalences of between 44% to 89% in sheep [33], 42% to 80% in goats [33] and 6% to 23% in pigs [18,34]. The seroprevalence rates in sheep and goats in the current study are lower than that previously reported on St. Kitts and Nevis [33]; however, the ages of animals in either study was not known and could be an influencing factor. Seroprevalence is known to increase with age [10] so it is possible the animals in the present study were younger than those sampled previously, leading to a lower seroprevalence. This may be likely given that the animals in the current study were sampled at abattoir and therefore processed for meat production, which normally occurs at a younger age. Worldwide seroprevalence in sheep and goats varies widely and is highly dependent on age of animal and the method of detection used; however, similar prevalences to those in this study have been reported in sheep and goats in South America [35,36], Central America [37], North America [38,39] and Europe [40,41]. Previous studies on St. Kitts demonstrated that domestic [20] and feral [21] cats on the island have some of the highest *T. gondii* seroprevalences ever reported, suggesting widespread contamination of the island with oocysts. Sporulated oocysts of *T. gondii* are very resistant to environmental conditions and can remain infective in moist soil or sand for up to 18 months [42] and in water for over a year [43]. They can then be a source of infection for ruminants if they contaminate vegetation in grazing areas or water supplies.

The higher seroprevalence rate in pigs in the present study may be a result of the wider range of transmission routes available to omnivores. Most pigs on St. Kitts and Nevis are reared outdoors where they are more likely to encounter oocysts shed by free-roaming cats and possibly also bodies of dead rodents and other small mammals harbouring *T. gondii* cysts in their tissues. Non-confinement housing is known to be a significant risk factor for *T. gondii* transmission [44,45] with seroprevalence rates in pigs in Europe declining over the years with the introduction of more intensive, indoor management systems [7]. The seroprevalence reported in the current study is similar to studies in South [46,47], Central [48] and North America [49], and Europe [50] where outdoor management systems were reported. In each species we studied, the serum ELISA and meat juice ELISA results correlated significantly. This is consistent with previous studies [51-53] and provides further evidence that meat juice samples can be used in seroprevalence studies where serum or plasma samples cannot be collected.

Detection of *T. gondii* DNA in tissue samples has been shown to be less sensitive than ELISA at detecting infection; however, in most studies, small samples of tissue were used for DNA extraction (0.2-1 g) [54,55]. We demonstrated a moderate correlation (*k* = 0.5204) between molecular and serological detection of infection but used far larger amounts of tissue (50 g) for DNA extraction. Since as few as one tissue cyst may be present in 50-100 g tissue [56], our method appears more likely to detect these low levels of infection.

Although our method appears more sensitive, 42 and 50 animals were positive by serum and meat juice ELISA, respectively, yet negative by qPCR. This discrepancy may have been due to the limited amount of sample processed. Although 50 g of tissue is significantly larger than the standard <1 g of tissue normally sampled for DNA extraction from meat, in most cases it did not constitute the whole heart so perhaps tissue cysts were missed during sampling. This is particularly true for the pigs, which may explain why a larger proportion of these animals were positive by ELISA but negative by qPCR. Choice of sample organ may also influence results. The brain is the most common site for *T. gondii* tissue cysts; however, collection of this organ was not a feasible option at the abattoir so the heart was chosen as this is also a commonly affected organ [57]. It was noted that 3 animals were positive by qPCR but negative by both serum and meat juice ELISAs. This may be because the animals were in the early stages of infection and although there was parasitemia, they had not yet seroconverted. Alternatively, these animals may have been harbouring a chronic infection and their antibody levels had waned [58].

Virtually all edible portions of an animal can harbour viable *T. gondii* tissue cysts [44] and although we did not test skeletal muscle for *T. gondii*, the high number of qPCR-positive animals in this study, and the fact that serologically positive animals can harbour tissue cysts for their lifetime, would suggest that people on St. Kitts and Nevis are at risk of infection by handling or consuming local sheep, goat or pig meat. Consumption of undercooked meat is a significant risk factor for infection [13], although the relative risk differs with cultural habits and geographical location. For example, 84% of pregnant women in Paris were found to have antibodies to *T. gondii* [44] which may be related to the propensity for French people to consume undercooked meat, particularly lamb [53]. Unfortunately, there is no data on the exposure of people on St. Kitts to *T. gondii*. In the Caribbean, meat is traditionally cooked very well and the risk of transmission by ingestion of viable tissue cysts is most likely low, since temperatures of over 67°C for 10 min will kill tissue cysts [59]. Infections are probably more likely to occur as a result of handling infected meat without subsequent hand washing and by ingesting oocysts on soiled hands or vegetables, or in contaminated water.

Genotyping of qPCR-positive DNA samples in the present study revealed a predominant Type III lineage, which is similar to previous data from the Caribbean. A study on feral cats on St. Kitts reported the presence of Type III genotypes as well as Type II and 2 unique genotypes [21]. Isolation and characterisation of *T. gondii* from free-roaming chickens in Grenada revealed that 29 out of 35 isolates were Type III, one was Type II and four were Type I [19]. In a subsequent study in Grenada, 1 out of 4 *T. gondii* isolates from mongooses was Type III and 3 were described as atypical genotypes [60]. In North America and Europe, Type II is the predominant lineage associated with opportunistic infections and congenital infections in humans [61,62], as well as infections in animals [32,63,64]. However, in South America, in particular Brazil, studies in free-roaming chickens have demonstrated that Type II is rare or absent and Type I, Type III and atypical genotypes dominate [65-67]. It is of note that atypical strains have been associated with severe and fatal toxoplasmosis in immune-competent patients in French Guiana [68] and Suriname [69].

Although we genotyped only a limited number of samples, we were able to demonstrate the presence of Type I genotypes in animals destined for the food chain. The Type I genotype is typically virulent in mice [70] and has been associated with reactivation of the parasite in immune-compromised individuals [71]. We also demonstrated possible re-assorted genotypes and mixed infections with alleles of Type II and Type I (10%) and alleles of Type II and Type III (10%). A previous study on feral cats in St. Kitts reported the presence of mixed infections (Type II and Type III alleles present across different loci) amongst 5 of 7 cats [21] demonstrating the possibility of infection with more than one genotype on the island. To confirm mixed infections in the current study, PCR products would have to be cloned and sequenced at different loci.

## Conclusions

In summary, the results of this study suggest widespread environmental contamination in St. Kitts and Nevis with *T. gondii* oocysts, and that livestock could be a potentially important source of *T. gondii* infection if their infected meat is consumed or handled undercooked. Although only a limited number of samples were genotyped, we demonstrated the presence of Type I genotypes in meat destined for human consumption which may have public health implications since Type I genotypes (or recombinants of Types I and III) have been suggested to be more likely to result in clinical toxoplasmosis [72]. Further studies into the genetic variation of *T. gondii* on St. Kitts are currently underway in our laboratory, and studies to determine the status of human infection in the region are warranted.
